# Radiation Dose Reduction in Different Digital Radiography Systems: Impact on Assessment of Defined Bony Structures in Bearded Dragons (*Pogona vitticeps*)

**DOI:** 10.3390/ani13101613

**Published:** 2023-05-11

**Authors:** Natalie Steiner, Eberhard Ludewig, Wiebke Tebrün, Michael Pees

**Affiliations:** 1 Department of Small Mammal, Reptile and Avian Diseases, University of Veterinary Medicine, 30559 Hanover, Germany; 2 Division of Diagnostic Imaging, Department of Small Animals and Horses, University of Veterinary Medicine, 1210 Vienna, Austria; 3 Wimex Agrarprodukte Import and Export GmbH, 93128 Regenstauf, Germany

**Keywords:** reptiles, lizards, digital radiography, image quality, dose reduction

## Abstract

**Simple Summary:**

Digital radiography has long been established in veterinary clinics, leading to increased use of digital systems in reptile species as well. In this study, we used different digital radiography systems on cadavers of bearded dragons (*Pogona vitticeps*). We aimed to examine the impact of a radiation dose reduction on the image quality and the assessment of defined skeletal structures. We employed a blinded assessment and a defined scoring system to evaluate the techniques tested. Our results demonstrate that both a 50% and a 75% reduction in the radiation dose significantly decreased image assessments. These findings highlight the need for correct radiation dose protocols to produce high-quality radiographs in reptile species.

**Abstract:**

Three different digital detector systems were used to study the effect of a defined radiation dose reduction on the image quality of digital radiographs in bearded dragons (*Pogona vitticeps*). A series of radiographs of seven bearded-dragon cadavers with a body mass ranging from 132 g to 499 g were taken in dorsoventral projection. The digital systems employed included two computed radiography systems (CR) (one system with a needle-based and one with a powdered-based scintillator) and one direct radiography system (DR). Three levels of the detector dose were selected: A standard dose (defined based on the recommended exposure value of the CR_P_, D/100%), a half dose (D/50%), and a quarter dose (D/25%). Four image criteria and one overall assessment were defined for each of four anatomic skeletal regions (femur, rib, vertebra, and phalanx) and evaluated blinded by four veterinarians using a predefined scoring system. The results were assessed for differences between reviewers (interobserver variability), radiography systems, and dosage settings (intersystem variability). The comparison of the ratings was based on visual grading characteristic (VGC) analysis. Dose reduction led to significantly lower scores in all criteria by every reviewer, indicating a linear impairment of image quality in different skeletal structures in bearded dragons. Scores did not differ significantly between the different systems used, indicating no advantage in using a computed or direct radiography system to evaluate skeletal structures in bearded dragons. The correlation was significant (*p* ≤ 0.05) for interobserver variability in 100% of the cases, with correlation coefficients between 0.50 and 0.59. While demonstrating the efficacy of the use of digital radiography in bearded dragons and the similar quality in using different computed or direct radiography systems, this study also highlights the importance of the appropriate level of detector dose and demonstrates the limits of post-processing algorithm to compensate for insufficient radiation doses in bearded dragons.

## 1. Introduction

Radiography in reptile medicine is an important diagnostic tool [1]. Various indications exist, such as suspected skeletal lesions, evaluation of the lungs, and changes in the gastrointestinal or reproductive tract [1,2,3,4,5,6]. Furthermore, radiography can also be helpful to assess the reproductive status or gender determination [7,8]. Due to their unique anatomy, reptile species often present some challenges for radiography such as small body sizes with miniature anatomical structures or the lack of internal fat tissue between organs and a lack of division between the thorax and abdomen, leading to limited soft tissue contrast. High-resolution screen film systems have been used to compensate to a certain degree for such limitations [1,2,9]. With the transition from screen film to digital radiography, reptile medicine was faced with the advantages and disadvantages of the new technology. Digital systems provide advantages such as a greater dynamic range, data transfer, a linear signal response, post-processing, and storage possibilities [9,10,11,12,13,14]. Even though screen-film systems have a superior spatial resolution, digital systems can differentiate very small attenuation differences better [12]. Various studies have already compared conventional to digital systems in reptiles, showing no less good image quality for diagnostic interpretation [9,12,15]. In contrast, digital images were superior for structures with high contrast such as bony structures and air–tissue boundaries as seen in the lungs [12]. Based on these results, the comparison of different digital systems amongst each other and the implementation of dose requirements for valuable diagnostic images is of great interest [11,13,16]. For a basic understanding, today, in veterinary medicine, different digital detector systems with different advantages are used: First, digital detector systems can further be divided into computed radiography (CR) and direct radiography (DR) [10,14,17,18]. DR includes flat panel systems, where a scintillator converts incoming X-rays directly into light [10,18]. CR uses storage phosphor image plates with a separate read-out process [14,18]. DR systems were long described as superior to phosphor storage panel images [19]. However, within the last 20 years, the basic principle of CR was modified to achieve both a higher detective quantum efficiency (DQE, the efficiency of a detector in converting X-ray energy into image signal) and spatial resolution. In particular, the introduction of needle phosphor plates (NIP) combined with a novel line-to-line CR stimulation and light collection technology improved the DQE of storage phosphor systems greatly [20,21,22]. This superiority can be used either for a higher signal-to-noise ratio (SNR) or dose reduction [13,21,22,23]. Wirth, S. et al. [19] even described a possible dose reduction in radiography of human skeletal structures of up to 75% with a NIP and a line-to-line scanner in comparison to flat panel systems and power-based phosphor detectors without inferior image quality. Concerning the importance of radiation exposure reduction for the patient and medical staff, the potential for dose reduction in digital systems seems therefore promising. In addition to the DQE in NIP systems, features such as post-image processing and the higher dynamic range in CR systems also reveal higher dose reduction potential than in DR systems. Unfortunately, even with the knowledge of dose reduction without the loss of image quality, taking this principle into clinical practice does show various difficulties. In digital radiography, there is no inverse correlation between dose and image contrast, therefore “film-blackening” as an indicator of overexposure no longer exists as seen in conventional radiography systems before. What generally can be seen when assessing digital radiography is a reciprocal relationship between the dose and the signal-to-noise ratio. However, the subjective and individual sensitivity to recognize an increase in noise is relatively low. Digital systems do therefore have a risk of substantially increasing the patient’s dose without even being aware of it. Furthermore, inadequate image processing or suboptimal image display also offers the potential for decreasing diagnostic information in digital radiography systems, leading to an increase in dose without the need for clinical evaluation [24]. Regarding the lack of subjective visual control ability of the optimal diagnostically relevant radiation dose, the implementation of dose indicators and dose monitoring is mandatory for digital radiography.

This study aimed to investigate the effect of a defined dose reduction for diagnostic imaging in skeletal structures of bearded dragons (*Pogona vitticeps*), regarding a possible loss in image quality, for three different digital detector technologies. The results could contribute to standardization in radiographic settings and dose control for reptile species. Regarding the importance of a reduction of radiation exposure for medical staff and the patient, the radiation dose should always be kept as low as possible, while achieving a diagnostically valuable image.

## 2. Materials and Methods

### 2.1. Radiographic Settings

For the radiographs, three different digital detector systems were used (Table 1).

Radiographs were taken using a bucky table unit (Philips Bucky Diagnost TH, Philips Healthcare, Hamburg, Germany). The animals were directly placed on the detector. An anti-scatter grid was not used. Exposure settings were adjusted on the base of the dose requirements of the CR_P_ system and evaluated for a reference animal (animal 1 in Table 2). Exposure settings of 42 kVp and 5 mAs were identified to generate images with a dose indicator value (lgM of 1.94) equivalent to a detector dose level of 2.5 µGy (D/100%).

The detector dose was subsequently reduced to half the dosage by halving the mAs value to 2.5 (D/50%) and to a quarter, quartering the mAs value to 1.25 (D/25%). Tube voltage was kept constant at 42 kVp for each image, leading to three different settings for each radiographic system (D/100% 42 kVp, 5 mAs, D/50% 42 kVp 2.5 mAs, D/25% 42 kVp 1.25 mAs). Dose-Area Product (DAP) measurements were performed for all systems to monitor the uniformity of exposure. The size of the exposed field of 20 × 30 cm^2^ and the focus-detector distance of 110 cm were kept constant for all animals and images. System-specific processing algorithms were used. In pre-studies, the parameters of these processing algorithms were evaluated regarding detail visibility.

### 2.2. Procedure

In this study, seven bodies of Inland Bearded Dragons (*Pogona vitticeps*) with a mean body mass of 292 g and a mean snout-to-vent length of 164 mm (Table 2) were used. Animal bodies were selected from patient animals that had to be euthanized due to various reasons not visibly affecting the skeletal system, with the owner’s agreement. Animals were euthanized and stored at −18 °C for different time periods between one and six months. All radiographs were taken only in the dorsoventral position. Evaluation of the radiographs was fully blinded, using a DICOM Anonymizer (https://sourceforge.net/projects/dicomanonymizer/, accessed on 10 July 2021) with randomly chosen unconnected four-digit numbers, not in any relation to the specific animal or exposure setting. Reviewers were four veterinarians with a minimum of two years of experience in a clinic specializing in reptile medicine and therefore regular training in the assessment of reptile radiographs. Assessments were conducted independently. The workstation was equipped with two medical grey-scale monitors (EIZO MX240W, matrix: 1920 × 1200 pixel, dot pitch: 0.27 mm; luminance: 320 cd/m^2^, contrast ratio: 850:1; Avnet Technology, Nettetal, Germany). Commercial medical image analysis software was used (GOP-View XR2-T, Contextvision, Stockholm, Sweden). To become familiar with the assessment system, prior to the study, the reviewers went through a training period evaluating 25 randomly chosen radiographs that were not used for the study results. Evaluation time per image was unlimited. The ambient light and other conditions of the viewing environment fulfilled the requirements for medical image interpretation.

### 2.3. Scoring System

For this study, exclusively skeletal structures were examined. Four different structures (femur, ribs, vertebra, and phalanx) were chosen based on different bone architecture or features such as the differentiation of bone to soft tissue and surroundings (Table 3). The femur was essentially chosen to evaluate the differentiation between corticalis and spongiosa, joint structures were evaluated based on the left front phalanx, and the last left rib was used to further evaluate the details and structure of the corticalis. For each anatomical structure, four different characteristics (Table 3) were evaluated using a four-scaled scoring system, ranging from 1 (optimal evaluation) to 4 (insufficient evaluation). Scoring systems were used according to the modification of Körner, M. et al. [25] (Table 4).

### 2.4. Statistical Analysis

In this study, four criteria were evaluated in seven animals using three different radiographic systems comparing three different dose levels, leading to 252 criteria evaluated by each of the four reviewers and resulting in 1008 examinations in total. Interobserver variability was addressed using Spearman’s rank correlation coefficient to test for possible differences between the reviewers themselves. Additionally, interobserver agreement was assessed using the Wilcoxon signed-rank test. Calculations were made using SPSS (IBM SPSS Statistics 20, IBM, Armonk, NY, USA). Mean values, scoring frequencies, and 95% confidence intervals were calculated for each criterion, and an overall assessment was conducted to facilitate comparison between systems and reviewers.

A visual grading characteristics (VGC) analysis was applied for intersystem comparisons. The obtained VGC curve graphically demonstrates the comparison of the two systems. In case of an equal rating, the curve would be diagonal resulting in an area under the curve (AUC) of 0.5. The more one system is rated superior, the more the curve moves to the respective axis, therefore changing the area under the curve value towards 0.0 or 1.0 [26]. Commercial software was used for the calculation of AUC and comparisons (Sigma Plot 11, Systat Software Inc., San José, CA, USA). For all calculations, the correlation was considered to be significant with *p* ≤ 0.05 and highly significant with *p* ≤ 0.001. The strength of the coefficient was assessed in accordance with recommended standards [27], with correlations between 0.1 and 0.3 considered low, correlations between 0.3 and 0.5 considered moderate, and over 0.5 considered high.

## 3. Results

### 3.1. Dose Effects

#### 3.1.1. Scoring of the Different Criteria

For all criteria in all systems, scores given by the reviewers were lower after the dose reduction, regardless of halving or quartering the full dose (D50% or D25%).

Scores regarding all criteria together were significant to highly significant, ranging from *p* = 0.008 to ≤0.001.

In all four criteria, comparisons of D25% to D100% reached significantly lower scores than the D50% to D100%. The only exception was the phalanx using the CR_P_, but this was still nearly significant. The ribs showed the least significant difference between scores after the dose reduction. In both CR systems, a significant difference in scoring was only seen after the dose reduction from D100% to D25%, and in the FP system, a reduction from D100% to D50% already scored significantly different. The vertebra always scored significantly less with decreasing dosages independently of the radiography system used. For the phalanx, a dose reduction led to significantly lower scores in all comparisons for FP and CR_N_, but using the CR_P_, no significant difference was seen comparing the different dosages used. The femur showed different results with each system used. The CR_N_ comparison of D50% to D25% showed no significant difference, while D100% to D50% and D100% to D25% did, with the latter even being highly significant. Using the CR_P_, only the comparison of D100% to D25% was significant, whereas while using the FP, every comparison of dosages showed a significant to highly significant difference in scoring (Table 5).

#### 3.1.2. Scoring of the Different Systems

Comparing the different radiography systems used, the FP system showed in all criteria, except one, significantly lower scores when reducing the dosage. Only for the criterion for the rib was the reduction from D50% to D25% not significant, but it was significant in the comparison of D100% to D50% and D100% to D25%. Regarding the CR_N_ system, only the criterion for vertebra showed significantly lower scores with every dose reduction. For the femur and the rib, only a reduction to a quarter of the dosage (D100% to D25%) showed a significantly lower score. For the criterion for the phalanx using the CR, none of the dosages compared showed any significant difference. The CR_N_ system showed significant differences in all reductions for the phalanx and vertebra but not for the femur, with no significant difference between D50% to D25%, and the ribs only showed significance in using a quarter of the dosage (D100% to D25%). Further details can be seen in Table 5.

#### 3.1.3. Comparison of the Different Systems

Comparisons of the different systems with each other were performed using the scoring of the overall assessment of the different criteria, while also assessing the three different dosages used (D100%, D50%, D25%). There was no significant difference between any of the systems with none of the dosages. AUC ranged between 0.50 and 0.59, with a mean of 0.55. Details can be seen in Table 6.

### 3.2. Interobserver Variability

The average scores given by the reviewers can be seen in Table 7 for each criterion and in Table 8 for each radiography system. Interobserver correlations were calculated for all criteria and overall assessments, as well as scoring between the different systems, adding up to 48 rank correlation values. Correlations were significant in 100% (48/48) of the cases and highly significant in 95.8% (46/48) of the cases. Reviewer 3 showed lower scores with a Spearman’s value of 0.33 and 0.38 in comparison to reviewers 1 and 2. Reviewer scores for the phalanx were again highly significantly correlated in all cases, with a Spearman’s value of 0.60 to 0.79, with a mean of 0.70. Scores for the ribs showed a Spearman’s value of 0.45 to 0.68, with a mean of 0.57, being highly significant in all the cases, and the vertebrae showed a Spearman’s value for reviewer’s scores of 0.45 to 0.68, with a mean of 0.62. The interobserver agreement for all criteria was highly significant in all cases. The agreements ranged from moderate (0.44) to high (0.66). Interobserver variability was also tested for the different systems, again showing highly significant correlations for every reviewer with each system. Spearman’s ranks ranged from 0.45 to 0.71 (average of 0.57) for CR_P_, from 0.38 to 0.68 (average of 0.54) for FP, and from 0.80 to 0.69 (average of 0.60) for CR_N_. Details on Spearman’s rank correlation can be found in Table 9 for each criterion and radiography system. Reviewers’ ratings were highly significantly different, except reviewer 4 compared to reviewer 1.

## 4. Discussion

### 4.1. General Aspects

Digital radiography systems have long been established in veterinary medicine and are therefore also used in exotic pet medicine. The awareness of the need for standardized protocols and dosage optimization has slowly begun to arise in the world of exotic pets. Even though many authors describe the use of radiography in reptiles [2,4,6,8,28], no to little information exists on technical settings to achieve images with optimal diagnostic value. The main conclusion of the present study is that a dose reduction in digital radiography systems may limit the image quality of skeletal structures in bearded dragons, and it is conceivable that this information can also cautiously be transferred to other small to medium lizard species. Assuming that the data in this study reflect real conditions such as in a clinical setting using living patients, a general dose reduction leads to decreased reviewer scores regarding image quality, although image processing algorithms might still produce a reasonable overall image impression.

### 4.2. Evaluation Methods

In the present study, different skeletal structures of varying thickness and structure were used to have a broader spectrum of differences such as the transition from spongiosa to corticalis (femur, criterion 1) or demarcation to the joint (phalanx, criterion 2). Visceral structures were not assessed due to the use of dead bodies and the general challenge of assessing visceral structures with digital radiography in reptiles [2,9]. Visual grading characterizing (VGC) analysis was used and is recommended to evaluate the performance of different radiography systems. By using visual grading, anatomical criteria can be evaluated objectively with a link to clinical interpretation. Still, bias can occur, in this case primarily regarding the reviewers rating the systems. The reviewers were chosen regarding their experience with radiography in reptile species with a minimum of two years of experience in regularly evaluating radiographs in a specialized clinic. A training session was conducted beforehand to reduce divergence in scoring and achieve standardization. However, scores did differ significantly from each other (except for reviewer 1 compared to 4), showing the importance of subjectiveness and individual experience by different human beings even when using a scoring system. The disadvantages and advantages of VGC analysis have already been described in other studies such as Tebrün, W. et al. [13], Båth, M. et al. [29], and Månsson, L. G. [30]. Despite a lack of agreement in scores, the tendency with which the reviewers did score the criteria was the same. Therefore, assessments were significantly correlated for all criteria and all reviewers. These correlations prove the validity of the method, as the interpretation of the study results exclusively relies on the comparison of the scores between the system and dosages.

### 4.3. Effect of Dose Reduction

Data showed that in every system, a dose reduction led to significantly worse scores for most of the criteria, especially in the “double” reduction from 100% to 25%. Regarding the criteria, the vertebra seems to be the most sensitive structure with significantly worse scores after every reduction with each system used. In contrast, the ribs showed the least influence of the dose reduction, only receiving decreased scores after a reduction of 25%. The effect of dose reduction for the criterion femur varied. In the ribs and femur, the reviewers had to evaluate the differentiation between the bone, whilst in the criteria for vertebra and phalanx, the demarcation to the surrounding tissue and joint space was addressed. The vertebra and phalanx are much more delicate structures than the femur and rib, indicating a possible greater impact of dose reduction on smaller structures. In particular, the joint space with a fine surface and superimpositions of other structures could therefore be more affected than more solid structures such as the femur. The ribs are much more delicate than the femur, impeding the evaluation of this criterion and possibly leading to a greater impact in dose reduction, again indicating a greater influence on smaller structures. In contrast, the femur showed the best scores, indicating that the thicker and bigger the structure, the higher the chance of being able to evaluate variances.

Reduced image quality with a decreased dosage results from a decreased signal-to-noise ratio. Noise is unavoidable in images produced by medical imaging, as no force distributes photons from the X-ray beam uniformly over the surface [31]. In digital radiography, the production of noise primarily depends on the elements of the radiography system, such as the detector, X-ray source, controller circuits, and others. Different techniques have been developed to reduce noise such as collimators or different types of filters [31]. Despite all these techniques, noise still exists and cannot be fully erased. Decreasing the dosage increases the effect of noise due to the lower number of photons emitted, leading to a higher signal-to-noise ratio. In this study, the dosage was decreased to half or a quarter of the reference dose. Studies such as Uffmann, M. et al. [24] described a low sensitivity of the human eye in knowingly detecting image noise, with complaints only after a 50% reduction in dosage. Therefore, the question arises as to whether diagnostic information is lost even before these reduction steps and if the human eye is a good evaluation tool for evaluating image quality, even though a dose reduction in digital radiography systems could be possible due to the higher detective quantum efficiency (DQE). Digital radiography systems, especially needle-based phosphorous systems, show a higher DQE than conventional screen film systems [21], allowing one to reduce the dosage while maintaining image quality.

### 4.4. Comparison of Detector Systems

In the present study, only digital radiography systems were used, as most veterinary practices today have changed from conventional to digital radiography. Three different systems were compared, namely one flat panel system (FP), a powder-based storage phosphor system (CR_P_), and a needle-based storage phosphor system (CR_N_), and as described above, they have individual pros and cons. Wirth, S. et al. [19] describes a better evaluation of bone structures in human limbs due to a clearer distinction of cortical structure, articular surface, and cortical delineation of the phalanx in CR_N_ systems compared to CR_P_ systems and FP systems with the possibility of a dose reduction of approximately 75% without a loss in image quality. This superiority of CR_N_ systems could not be reproduced in our study when assessing the skeletal structures in bearded dragons. Our results showed no significant difference in scores between the systems. There was a tendency for better performances in the CR_N_ systems compared to the others, but with no significance. The flat panel system seemed to be the most sensitive system with worsening scores after every dose reduction, again with no significant difference between the other systems. The reason for this discrepancy can possibly be found in the use of a small reptile species, with structures even smaller and finer than in the phalanx of the human skeletal system. On such small levels, the benefits of CR_N_ systems over the others could be nullified, leading to similar image quality in all systems used.

In general, it was not the aim of the study to validate the different technical systems, but rather only to assess the impact of dose reduction in each system. Therefore, with this study design and focused on the skeletal system in bearded dragons, we only conclude that all three systems seem to produce diagnostically reliable images with a possible beneficial use for CR_N_ systems.

### 4.5. Limitations of the Study

The study was conducted using dead animals, therefore only skeletal structures were chosen for assessment. No conclusion can be drawn regarding soft tissue representation with decreased radiation dosages. Using dead animals also leads to a lack of movement due to respiration or heart action, which must be kept in mind as it could have a possible impact on image quality in living animals. Soft tissue structures are surely more prone to respiration artifacts than skeletal structures, but overall influence could still be possible. Even though only animal bodies without detectable abnormalities in the bony structures were used, the animals were not undergoing specific tests regarding the underlying disease. The underlying disease could individually affect bone density or shape and can therefore not be fully ruled out. Regarding the different animals, it has to be mentioned that the size ranged from 134 to 499 g. We see this variation as minor as all the results point in the same direction and this body mass range also presents the typical sizes of bearded dragons presented in practice. Due to the limited number of individuals, we did not calculate statistical evaluation depending on the size, but this could be the focus of further studies. The duration of freezing after euthanasia varied from one to six months and could theoretically lead to different stages of decomposition of the animal body. As we only selected skeletal structures, we expect only a small impact on bone structure due to freezing, making this a minor limitation, which still should be kept in mind, regarding future studies. Reviewer numbers were rather low, allowing a higher risk for variation between the observations. Only one species and a limited number of animals were used, although the overall number of assessments allowed for detailed statistical comparisons. This study was conducted using only bearded dragons. Translation of the results to other reptile species should be made cautiously as reptile species vary greatly in size, shape, and even anatomical structures. Nevertheless, bearded dragons were chosen due to the high popularity of this species in European countries and therefore the higher clinical impact than rarer species. Further studies should go into detail comparing delicate structures in different settings to provide more in-depth information, as well as extend to the use of different species. Finally, smaller steps in dose reduction could perhaps allow for more detailed results. Additionally, the scales in reptiles could influence image quality and further aggravate the possibility of dose reduction while maintaining image quality [9].

## 5. Conclusions

The study results demonstrate that with digital radiography systems, an optimal dosage according to the needs of the digital system is essential. A general dose reduction is not recommended in reptile species as it will cause a loss of clinically relevant information even if the image quality subjectively appears sufficient. Further studies with smaller steps in dosage reduction should be conducted, as well as methods such as using artificial intelligence to replace the high subjectiveness of the reviewers and insensitivity of the human eye. This study shows the highly difficult aspect of defining a minimal dose to reliably answer specific diagnostic questions, regarding the vast variety of influences on image quality such as patient-related, system-related, and observer-related factors.

## Figures and Tables

**Table 1:** Technical equipment and exposure settings used in the experimental setup. Al: Aluminum equivalent.

System	X-ray System	Detector System	Exposure Setting
FP—100%	PHILIPS Bucky Diagnost TH Grid: no Focus-Detector Distance 110 cm Focus size: 0.6 × 0.6 mm^2^ Filtration: 2.5 mm Al	TRIXELL Digital flat-panel detector 4343 RC-E (Detector size: 43 × 43 cm^2^)	42 kVp, 5 mAs
FP—50%			42 kVp, 2.5 mAs
FP—25%			42 kVp, 1.25 mAs
CR_P_—100%		FUJI HR/PHILIPS AC 500 (Screen size: 18 × 24 cm^2^)	42 kVp, 5 mAs
CR_P_—50%			42 kVp, 2.5 mAs
CR_P_—25%			42 kVp, 1.25 mAs
CR_N_—100%		AGFA DX-S (Screen size 18 × 24 cm^2^)	42 kVp, 5 mAs
CR_N_—50%			42 kVp, 2.5 mAs
CR_N_—25%			42 kVp, 1.25 mAs

**Table 2:** Weight and snout-to-vent length of animals used in the study. Animals are listed in order of radiographic examination.

Number of the Animal	Weight [g]	Snout to Vent Length [mm]
1	499	237
2	397	208
3	334	214
4	318	190
5	132	147
6	198	160
7	164	155

**Table 3:** Definition of criteria for radiographic assessment.

Criterion	Description
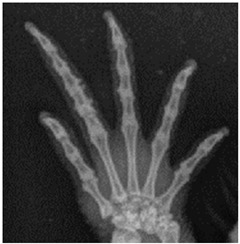	Left phalanx of the forelimb: identifiability of the joint contours of the interphalangeal joints: visualization and demarcation of the bone contour to the joint space, demarcation to the surrounding tissue.
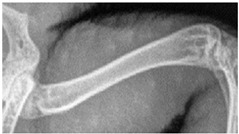	Right femur: assessability of the trabeculae and cortical bone: etail of the trabeculae and cortical bone, delineability of the structures from the surrounding area.
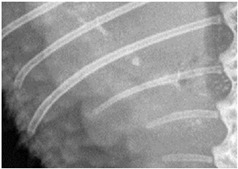	Left last rib: assessability of cancellous bone and cortical bone along the bone:demarcability of cancellous bone to cortical bone, demarcability of structures to surrounding area.
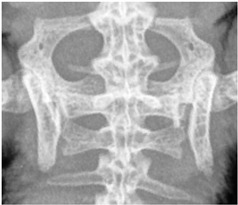	Cruciate vertebrae, 1st–3rd caudal vertebrae: identifiability of the individual vertebral bodies:assessability of the architecture and external contour of the vertebral bodies, delineability of the vertebrae from each other and from the surrounding area.

**Table 4:** Definition of the scores for radiographic assessment.

Scoring	Assessment
1	Optimal impression, structure completely evaluable, no limitation for clinical interpretation.
2	Good impression, structure evaluable, minor limitation for clinical interpretation.
3	Acceptable impression, detail representation limited, clinical interpretation restricted.
4	Insufficient impression, no interpretation possible.

**Table 5:** Summary of the statistical analyses stating significant occurrence in interobserver variability through statistically calculated AUC (area under the curve) values. CR_N_ = needle-based detector system, CR_P_ = powder-based detector system, FP = flat panel detector system. For interpretation of the AUC values see Section 2.4.

System	Criterion	Dose Comparison	AUC	95% Confidence Interval	*p*-Value
CR_N_	Femur	100–50	0.727	0.595–0.859	0.004
		100–25	0.811	0.700–0.923	<0.001
		50–25	0.630	0.484–0.776	0.095
	Phalanx	100–50	0.678	0.536–0.820	0.022
		100–25	0.781	0.657–0.906	<0.001
		50–25	0.653	0.509–0.797	0.049
	Rib	100–50	0.645	0.500–0.791	0.062
		100–25	0.773	0.647–0.899	<0.001
		50–25	0.635	0.489–0.781	0.082
	Vertebra	100–50	0.695	0.555–0.834	0.012
		100–25	0.833	0.724–0.942	<0.001
		50–25	0.662	0.520–0.805	0.037
	All	100–50	0.683	0.613–0.753	<0.001
		100–25	0.796	0.737–0.855	<0.001
		50–25	0.645	0.573–0.717	<0.001
CR_p_	Femur	100–50	0.591	0.441–0.741	0.241
		100–25	0.730	0.594–0.866	0.003
		50–25	0.651	0.506–0.796	0.052
	Phalanx	100–50	0.617	0.470–0.764	0.132
		100–25	0.651	0.507–0.794	0.053
		50–25	0.528	0.376–0.681	0.719
	Rib	100–50	0.557	0.405–0.708	0.466
		100–25	0.679	0.539–0.819	0.022
		50–25	0.617	0.470–0.765	0.132
	Vertebra	100–50	0.667	0.523–0.810	0.032
		100–25	0.771	0.645–0.897	<0.001
		50–25	0.656	0.511–0.800	0.046
	All	100–50	0.605	0.531–0.678	0.007
		100–25	0.701	0.633–0.770	<0.001
		50–25	0.603	0.529–0.677	0.008
FP	Femur	100–50	0.746	0.618–0.874	0.002
		100–25	0.851	0.748–0.954	<0.001
		50–25	0.659	0.516–0.803	0.041
	Phalanx	100–50	0.673	0.531–0.815	0.026
		100–25	0.823	0.714–0.931	<0.001
		50–25	0.709	0.573–0.845	0.007
	Rib	100–50	0.682	0.541–0.824	0.019
		100–25	0.771	0.643–0.899	<0.001
		50–25	0.621	0.474–0.769	0.120
	Vertebra	100–50	0.702	0.562–0.843	0.009
		100–25	0.790	0.670–0.911	<0.001
		50–25	0.680	0.536–0.824	0.021
	All	100–50	0.699	0.630–0.768	<0.001
		100–25	0.803	0.745–0.861	<0.001
		50–25	0.661	0.590–0.732	<0.001

**Table 6:** Summary of the statistical analyses stating significant occurrence in intersystem variability through statistically calculated AUC values. CR_N_ = needle-based detector system, CR_P_ = powder-based detector system, FP = flat panel detector system. For interpretation of the AUC values see Section 2.4.

	Dose Comparison	AUC	95% Confidence Interval	*p*-Value
CR_N_ vs. CR_P_	100%	0.556	0.403–0.708	0.476
	50%	0.540	0.388–0.693	0.606
	25%	0.541	0.388–0.693	0.600
CR_N_ vs. FP	100%	0.547	0.394–0.700	0.550
	50%	0.581	0.430–0.732	0.298
	25%	0.589	0.438–0.741	0.251
CR_P_ vs. FP	100%	0.496	0.343–0.649	1.039
	50%	0.537	0.385–0.690	0.635
	25%	0.549	0.397–0.700	0.534

**Table 7:** Average scoring from all reviewers for the different criteria and radiography systems. CR_N_ = needle-based detector system, CR_P_ = powder-based detector system, FP = flat panel detector system.

System	Dose	Femur	Phalanx	Rib	Vertebra
CR_P_	100	2.04	2.18	2.39	2.29
	50	2.25	2.61	2.61	2.82
	25	2.64	2.71	3.07	3.25
CR_N_	100	1.89	2.18	2.03	2.11
	50	2.54	2.71	2.82	2.71
	25	2.86	3.14	3.29	3.21
FP	100	1.82	2.11	2.18	2.29
	50	2.50	2.75	2.86	2.96
	25	2.89	3.36	3.25	3.39

**Table 8:** Average scoring of each reviewer for the different radiography systems used. Mean as average for all reviewer scores. CR_N_ = needle-based detector system, CR_P_ = powder-based detector system, FP = flat panel detector system.

Radiography System	Reviewer 1	Reviewer 2	Reviewer 3	Reviewer 4	Mean
CR_P_	2.50	2.37	2.87	2.55	2.57
CR_N_	2.85	2.29	2.76	2.70	2.65
FP	2.68	2.35	3.10	2.67	2.70

**Table 9:** Spearman’s rank correlation coefficient comparing each reviewer with each other regarding scoring of the different criteria and radiography systems used. * showing significant correlation; ** showing highly significant correlation (*p* ≤ 0.001). CR_N_ = needle-based detector system, CR_P_ = powder-based detector system, FP = flat panel detector system.

	Comparison between Reviewers					
	1–2	1–3	1–4	2–3	2–4	3–4
Overall	0.645 **	0.490 **	0.663 **	0.438 **	0.511 **	0.615 **
Femur	0.629 **	0.330 *	0.624 **	0.375 *	0.526 **	0.428 **
Phalanx	0.707 **	0.595 **	0.753 **	0.655 **	0.793 **	0.667 **
Rib	0.680 **	0.533 **	0.622 **	0.454 **	0.473 **	0.679 **
Vertebra	0.741 **	0.571 **	0.754 **	0.495 **	0.608 **	0.543 **
CR_P_	0.631 **	0.487 **	0.711 **	0.453 **	0.526 **	0.622 **
CR_N_	0.684 **	0.586 **	0.640 **	0.479 **	0.501 **	0.690 **
FP	0.679 **	0.479 **	0.650 **	0.379 **	0.518 **	0.557 **

## Data Availability

The data are provided as an online supplement to this article, https://doi.org/10.17605/OSF.IO/8H956, last accessed on 15 April 2023.
